# Disrupted epithelial/macrophage crosstalk via Spinster homologue 2-mediated S1P signaling may drive defective macrophage phagocytic function in COPD

**DOI:** 10.1371/journal.pone.0179577

**Published:** 2017-11-07

**Authors:** Hai B. Tran, Hubertus Jersmann, Tung Thanh Truong, Rhys Hamon, Eugene Roscioli, Miranda Ween, Melissa R. Pitman, Stuart M. Pitson, Greg Hodge, Paul N. Reynolds, Sandra Hodge

**Affiliations:** 1 Lung Research Unit, Hanson Institute and Department of Thoracic Medicine, Royal Adelaide Hospital, and Department of Medicine, University of Adelaide, Adelaide, Australia; 2 Department of TB & Lung Diseases, Hospital 175, Hochiminh City, Vietnam; 3 Centre for Cancer Biology, University of South Australia and SA Pathology, Adelaide, Australia; National Yang-Ming University, TAIWAN

## Abstract

**Introduction:**

We have previously established a link between impaired phagocytic capacity and deregulated S1P signaling in alveolar macrophages from COPD subjects. We hypothesize that this defect may include a disruption of epithelial-macrophage crosstalk via Spns2-mediated intercellular S1P signaling.

**Methods:**

Primary alveolar macrophages and bronchial epithelial cells from COPD subjects and controls, cell lines, and a mouse model of chronic cigarette smoke exposure were studied. Cells were exposed to 10% cigarette smoke extract, or vehicle control. Spns2 expression and subcellular localization was studied by immunofluorescence, confocal microscopy and RT-PCR. Phagocytosis was assessed by flow-cytometry. Levels of intra- and extracellular S1P were measured by S1P [3H]-labeling.

**Results:**

Spns2 expression was significantly increased (p<0.05) in alveolar macrophages from current-smokers/COPD patients (n = 5) compared to healthy nonsmokers (n = 8) and non-smoker lung transplant patients (n = 4). Consistent with this finding, cigarette smoke induced a significant increase in Spns2 expression in both human alveolar and THP-1 macrophages. In contrast, a remarkable Spns2 down-regulation was noted in response to cigarette smoke in 16HBE14o- cell line (p<0.001 in 3 experiments), primary nasal epithelial cells (p<0.01 in 2 experiments), and in smoke-exposed mice (p<0.001, n = 6 animals per group). Spns2 was localized to cilia in primary bronchial epithelial cells. In both macrophage and epithelial cell types, Spns2 was also found localized to cytoplasm and the nucleus, in line with a predicted bipartile Nuclear Localization Signal at the position aa282 of the human Spns2 sequence. In smoke-exposed mice, alveolar macrophage phagocytic function positively correlated with Spns2 protein expression in bronchial epithelial cells.

**Conclusion:**

Our data suggest that the epithelium may be the major source for extracellular S1P in the airway and that there is a possible disruption of epithelial/macrophage cross talk via Spns2-mediated S1P signaling in COPD and in response to cigarette smoke exposure.

## Introduction

Chronic obstructive pulmonary disease (COPD) is a common lung disease strongly associated with chronic exposure to inhaled irritants, especially cigarette smoke. The third most common cause of death worldwide, the disease remains incurable using current therapeutic interventions and identification of new therapeutic targets presents both an urgent need for health services and challenging tasks for translational researchers [1]. We have pioneered the pathological concept of defective phagocytosis by alveolar macrophages as a potential contributor to the pro-inflammatory cellular milieu in COPD and other chronic inflammatory diseases of the airway [2–5]. Defective phagocytosis of apoptotic bodies (efferocytosis) in the airway leads to their accumulation and potential secondary necrosis with ensuing inflammation that cannot be resolved even after smoking cessation [6]. Further to this, defective phagocytosis of microorganisms in COPD may contribute to airway colonization with potentially pathogenic microbes which contribute to increased risk for exacerbations and further inhibition of efferocytosis [7]. Importantly, our studies support a biological paradigm that macrophage phagocytic function can be regulated for therapeutic gain [8–10]. For example, low-dose azithromycin therapy was shown to significantly improve the phagocytic function of alveolar macrophages *in vitro* and in a human phase II study of COPD subjects [8, 10]. The precise mechanisms for the defective phagocytic function however, remain elusive.

Sphingosine-1-phosphate (S1P), sphingosine and ceramides are lipid mediators that regulate a range of vital cellular functions including cell death, survival/growth, motility and migration. This is achieved by the so called sphingolipid ‘rheostat’ which represents a complex balance of enzymes and proteins involved in metabolism, transport, and signal transduction of sphingolipid mediators [11]. S1P in particular directs leukocyte egress from lymphoid tissues and ingress into destination tissues, governs angiogenesis and various morphogenetic processes [12]. Our previous studies have shown that human lung tissue and alveolar macrophages demonstrate a complex expression profile for the individual components of the S1P signaling system, including synthesizing and degrading enzymes and receptors which undergo significant changes in COPD [9, 13, 14]. The complexity of the S1P signaling system was highlighted by our studies using cigarette smoke-exposed THP-1 macrophages [13, 14]. Thus, although cigarette smoke extract upregulated gene expression of Sphingosine kinases (SPHK1/2, enzymes responsible for S1P synthesis) and inhibited phagocytosis, (a feature observed also by selective inhibition of SPHK1/2), the activities of these enzymes were reduced in parallel with protein dislocation from their normal subcellular localization [14]. Furthermore, whilst stimulatory effect on phagocytosis by exogenous S1P indicated that signaling via S1P receptors (S1PRs)) is required for maintaining phagocytic function and that this could be deficient in cigarette smoke-exposed macrophages [14], cigarette smoke was also shown to significantly increase levels of S1PR2/5 transcripts [13]. Thus, translation of the S1P signaling system as a novel target in macrophage-based therapeutic approach in COPD requires more research to dissect its mechanism.

S1P is exported from the cell to deliver autocrine/paracrine effects via G-protein-coupled S1PRs on the cell surface [12]. Spinster homolog 2 (Spns2) has been recently identified as a transporter protein for S1P [15]. We therefore hypothesised that the bronchial epithelium could be the major cell type producing extracellular S1P in the airway, and that the inhibitory effects of cigarette smoke on macrophage phagocytic function may include disruption of the epithelial-macrophage crosstalk via intercellular S1P signaling involving Spns2. We comparatively analyzed S1P and the expression and localization of Spns2 in primary bronchial epithelial cells and macrophages from COPD vs. control subjects, chronically cigarette smoke-exposed mice and cigarette smoke-exposed THP-1 macrophages, and investigated the association with macrophage phagocytic function.

## Materials and methods

### Antibodies

Unless specified, the primary antibody to Spns2 was a goat polyclonal antibody directed against an intracellular domain (G-14, Santa Cruz, Dallas, TX, USA). In additional experiments, rabbit polyclonal antibodies directed against the aa71-120 domain (LifeSpan BioSciences, Seattle, WA, USA) or the N-terminus (GeneTex, Irvin, CA, USA) were used to confirm similar patterns of immunofluorescence. Rabbit SPHK1 and SPHK2 polyclonal antibodies were from Bioss (Woburn, MA, USA). Beta-actin mouse monoclonal antibody was from Sigma-Aldrich (St. Louis, MO, USA). PAN (rabbit anti-cytokeratin) antibody was from Invitrogen (Carlsbad, CA, USA). Secondary donkey IgG F(ab’)2 fragment antibodies conjugated to AF488, AF594 or AF647, specific to rabbit, goat, and mouse IgG (respectively) were all from Jackson ImmunoResearch (West Grove, PA, USA).

### Subject population

Seventeen donors, including 8 healthy never smokers, 4 non-smoker lung transplant recipients with stable lung function, and 5 current smokers of whom 4 diagnosed with COPD, were recruited in this study for broncho-alveolar lavage (BAL) during bronchoscopy (data in S1 Table). During bronchoscopy bronchial epithelial cells were also obtained via bronchial brushing in 3 nonsmoker subjects. COPD status was defined based on GOLD criteria (dyspnea and chronic cough or sputum production, history of cigarette smoking, and lung function tests FEV1/FVC<0.7). Nasal epithelial brushings were obtained from 2 healthy never smokers. Lung and nasal samples were obtained according to protocols approved by the Royal Adelaide Hospital Ethics Committee. Written informed consent was obtained from each donor. Subjects found with positive bacterial cultures from samples were excluded.

### Preparation of samples

Purification of alveolar macrophages from BAL by adherence to plastic was carried out as previously described [2]. Bronchial epithelial cells were directly prepared into cytospins. Nasal epithelial cells were collected and grown in submerged culture according to previously published protocols [16]. Alveolar macrophages from 4 non-smoker transplant patients and 2 healthy controls, and nasal epithelial cells from 2 healthy donors were treated for 24h with 10% cigarette smoke extract, or vehicle, as previously described [9].

### Human cell lines

THP-1 monocytes (ATCC, Manassas, VA, USA), and 16HBE14o- cells (a kind gift from Dr Dieter Guenter) were used. Their culture, differentiation of monocytes to macrophages with PMA, and exposure of cells to 10% cigarette smoke extract were carried out as previously described [9, 14].

### Mouse model of chronic exposure to cigarette smoke

Six mice exposed to cigarette smoke for six weeks and 6 ‘sham-exposed’ control mice were used as previously described [17, 18] in protocols approved by the Institute of Medical and Veterinary Science Animal Ethics Committee. Mice were humanely killed in accord with previous protocol using an overdose of ketamine/xylacine and careful cervical dislocation. One lung from each mouse was disaggregated to a cell suspension using a Medimachine (BD Australia, North Ryde, NSW, Australia), and macrophages obtained by adherence to a plastic surface. The phagocytic capacity of macrophages towards apoptotic 16HBE14o- cells and nontypeable *Haemophilus influenzae* (NTHi) was performed using flow cytometry as described [17–19]. The remaining lung was processed into a paraffin block.

### Immunofluorescence and confocal microscopy

Immunofluorescence of cytospins and chamberslide cell preparations were carried out as previously described [14]. Mouse lung tissue blocks were cut into 5 um sections which were microwaved for antigen retrieval and immunofluorescence staining as previously described [20]. Briefly, cells and tissues were allowed to bind primary antibodies for 16–18 h at 4°C, and secondary antibodies during a further 1 h at room temperature, with repeated washing applied after each incubation. Images were taken from at least nine optical fields (cells) or bronchioles (tissue) using a laser confocal microscope (LSM700, Carl Zeiss Australia, North Ryde, NSW, Australia). Non-bias selection and focusing was undertaken by using the DAPI channel. Quantitative analysis of immunofluorescence intensity was carried out using ImageJ software (NIH, Bethesda, MD, USA) [14]. Alveolar macrophages and type 2 alveolar cells in mouse lung sections were identified by their localization and morphology, a method justified in our previous study by differential expression of F4/80 in macrophages and SP-D in type 2 alveolar cells [20].

### RT-PCR

Extraction of total RNA from THP-1 macrophages, reverse transcription, and quantitative real-time PCR for measurement of Spns2 gene expression were carried out as previously described [9].

### Determination of S1P in cigarette smoke-exposed THP-1 macrophages and primary nasal epithelial cells

Passage 1–2 primary human nasal epithelial cells were seeded into 6-well collagen coated plates for a density the next day of 75% confluence, and treated with 10% cigarette smoke extract for 16 h in serum-free media (Bronchial Epithelial Growth Media, Lonza Pty Ltd. Australia, Mt Waverly, VIC) supplemented with 2% of Ultroser G Serum Substitute (Pall Corporation, Cerga-Saint-Christophe, France). To measure the rate of S1P formation in intact cells and the media an *in situ* assay was performed as previously described [21]. Briefly, cells were labelled with 0.5 μCi of [^3^H]-sphingosine (Perkin-Elmer) per well. After 30 min incubation at 37°C in a humidified atmosphere of 5% CO_2_, the conditioned medium was removed and kept on ice and the cells were scraped into cold PBS and pelleted at 500 g for 5 min. [^3^H]-S1P formed during the 30 min incubation was then extracted from both the conditioned medium and cells via a modified Bligh-Dyer extraction. Cell pellets were re-suspended in 300 μL of acidified methanol (100:1, methanol: concentrated HCl) and the suspension was sonicated for 30 seconds in a bath sonicator. Following this 300 μL of 2M KCl, 300 μL of chloroform, and 30 μL of 3M NaOH were added. Samples were centrifuged at 13,000 g for 5 min to separate phases and [^3^H]-S1P in the upper aqueous methanol phase (separated from sphingosine in the lower chloroform phase) was then analyzed by scintillation counting. Extracellular [^3^H]-S1P in the conditioned medium was extracted in a similar manner with 1 mL of methanol, 1 mL of chloroform, and 100 μL of 3M NaOH added to 1 mL of conditioned medium. Phases were separated by centrifugation at 1,000 g for 5 min and [^3^H]-S1P in the upper aqueous methanol phase was then analyzed by scintillation counting.

### Statistical analysis

Differences between groups were assessed using Kruskal–Wallis and Mann–Whitney *U*-tests, or Wilcoxon signed ranks test as appropriate, and SPSS software (SPSS Inc., Chicago, IL, USA). Associations between data were performed using Pearson’s correlation. *P* < 0.05 considered significant.

## Results

### Spns2 expression and subcellular localization in human alveolar macrophages

**Fig 1:**
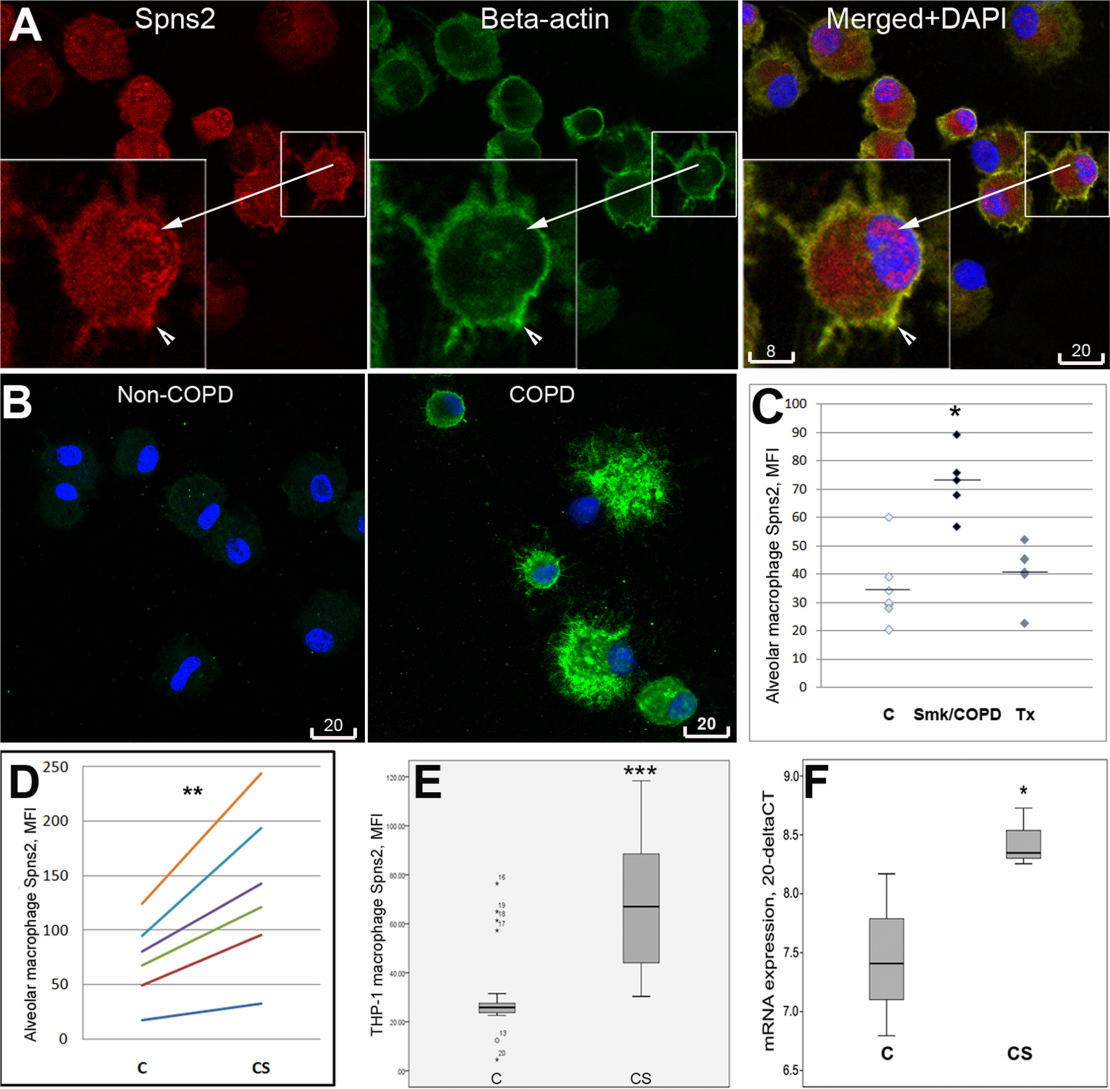
Spns2 expression in human macrophages. **A**, Expression and subcellular localization of Spns2 in alveolar macrophages. Spns2 (rabbit antibody) was labeled in red, beta-actin in green, blue was staining of nuclei. Spns2 was localized to plasma membrane (arrowhead), nucleus (arrow), and cytoplasm. **B**, Representative confocal images of Spns2 (goat antibody, green) in COPD vs. non-COPD alveolar macrophages, captured for quantitative analysis. For the highly intense Spns2 immunofluorescence in the given COPD sample not become saturated, the laser settings had to be set as low as the immunofluorescence in the non-COPD sample was nearly invisible for naked eyes. Scale bars in **A** and **B** are in micrometers. **C**, Significant increase of Spns2 immunofluorescence in alveolar macrophages of smokers/COPD patients (n = 5) compared to healthy control (C, n = 8, p<0.01) and non-COPD non-smoker transplant patients (Tx, n = 4, p<0.01). **D**, Cigarette smoke extract induced significant increase (**, p<0.01) of Spns2 immunofluorescence in primary alveolar macrophages obtained from non-smokers (n = 6). **E**, Cigarette smoke extract induced significant increase (***, p<0.001, 5 experiments) of Spns2 immunofluorescence in THP-1 macrophages. **F**, Cigarette smoke extract induced significant increase (*, p<0.05, 3 experiments) of Spns2 mRNAs in THP-1 macrophages.

We firstly examined protein expression and localization of Spns2 in BAL alveolar macrophages of healthy and non-COPD patient donors. Weak or moderate immunofluorescence staining of Spns2 was observed near the plasma membrane and remarkable staining of nuclei and cytoplasm was also detected (Fig 1A). As the localization and biologic function of Spns2 in the nucleus not been described previously, we performed a bioinformatics analysis using the online NLS-Mapper software (http://nls-mapper.iab.keio.ac.jp/cgi-bin/NLS_Mapper_form.cgi) on *Spns2* gene primary sequence and determined a predicted bi-partile Nuclear Localization Signal (data in S1 Text).

Alveolar macrophages obtained from COPD patients in this study showed very bright Spns2 immunofluorescence. Thus, to avoid immunofluorescence saturation in photography of COPD samples, confocal laser and gain settings had to be reduced resulting in some control samples appearing nearly invisible to naked eyes (Fig 1B). Despite the small size, this study revealed a significant increase in Spns2 immunofluorescence in COPD alveolar macrophages compared to those from healthy donors or lung transplant patient controls (Fig 1C).

### Spns2 expression in cigarette smoke-exposed macrophages

Primary alveolar macrophages and THP-1 macrophages were exposed to cigarette smoke extract *in vitro*. In both models, cigarette smoke extract induced a statistically significant increase of Spns2 immunofluorescence (Fig 1D and 1E). Upregulated expression at the mRNA level was also demonstrated in cigarette smoke-exposed THP-1 macrophages (Fig 1F).

### Spns2 and SPHK1/2 expression by primary bronchial epithelial cells

**Fig 2:**
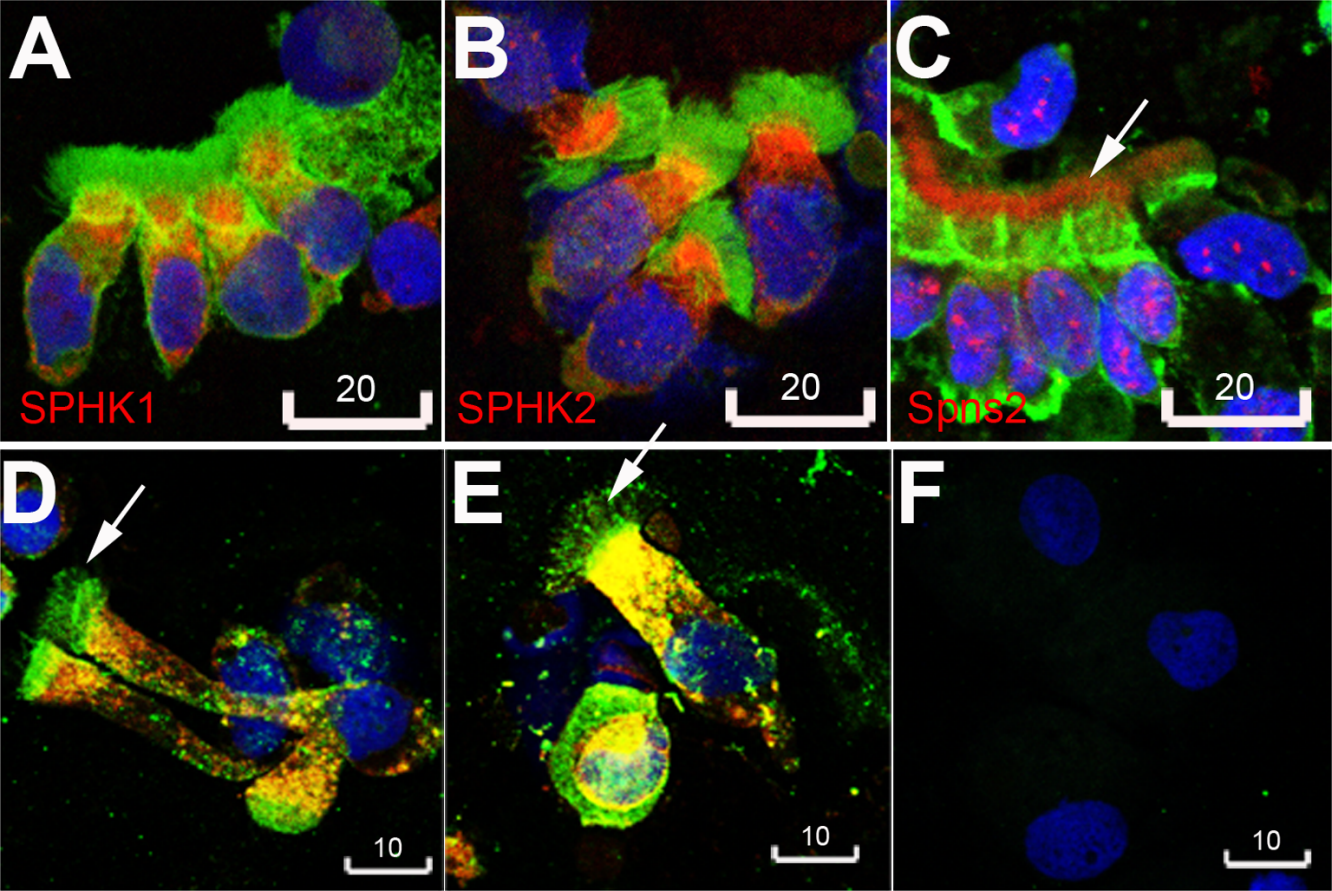
Human bronchial epithelial cells abundantly express SPHK1, SPHK2 and Spns2. **A,** SPHK1 (red). **B**, SPHK2 (red). **C**, Spns2 (red, LifeSpan BioSciences rabbit polyclonal antibody). Green in **A-C** was beta-actin. **D, E**, Spns2 (green, Santa Cruz goat polyclonal antibody) was expressed in cilia, and colocalized with SPHK1 (red, yellow being merged color of red and green) in the cytoplasm. **F**, a negative staining control incubated with conjugated antibodies alone. Blue in **A-F** was DAPI. Arrows in **C, D, E** indicate Spns2 expression in cilia. Scale bars are in micrometers. Images are representative of bronchial epithelial cells obtained from 3 different non-smoking donors.

In cytospins of primary bronchial epithelial cells obtained from 3 healthy control donors, bright immunofluorescence of SPHK1, SPHK2, and Spns2 was demonstrated (Fig 2). Spns2 was immunolocalized by two different antibodies to cytoplasm and nucleus, and of note, the apex was defined as harboring a large amount of Spns2 which was localized within the cilia (Fig 2C, 2D and 2E).

### Spns2 expression in cigarette smoke-exposed epithelial cells

**Fig 3:**
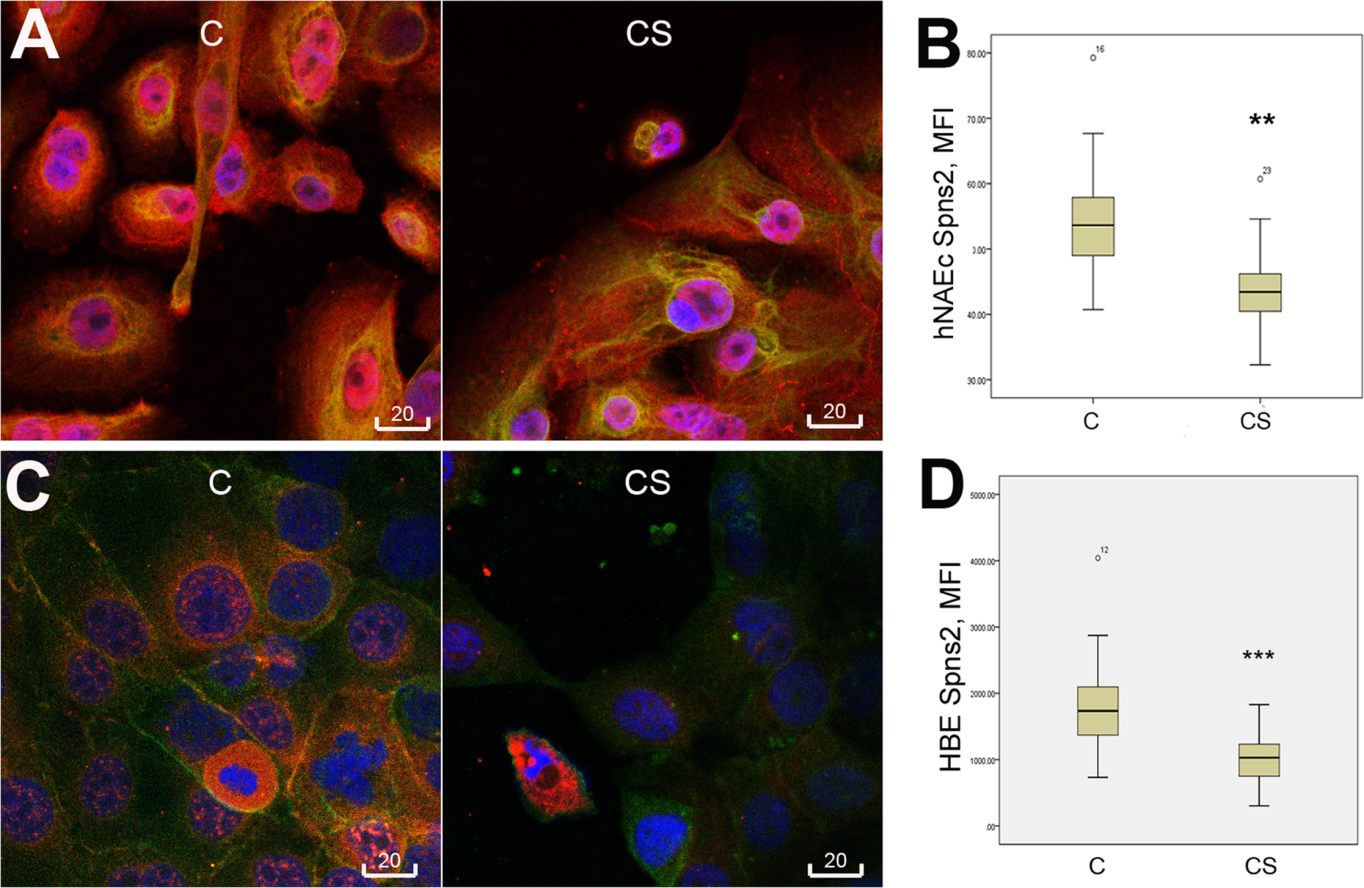
Cigarette smoke extract induced decrease of Spns2 expression in epithelial cells. **A**, Representative confocal images of Spns2 in primary cultures of human nasal epithelial cells (hNAEc), cigarette smoke extract-treated (CS) vs. control (C). **B**, Measurement of Spns2 mean fluorescence intensity (MFI) in hNAEc; **, p = 0.001 significant reduction in CS-treated cells, data pooled from 2 experiments. **C**, Representative confocal images of Spns2 in 16HBE cell line, treated with CS vs. control. **D**, Measurement of Spns2 mean fluorescence intensity (MFI) in 16HBE cells; ***, p<0.001 significant reduction in CS-treated cells, data pooled from 2 experiments. Red: Spns2; Green: cyto-keratin (epithelial-specific marker) in **A**, and β-actin in **C**. Scale bar = 20μm.

We next investigated a possible effect of cigarette smoke exposure on expression of Spns2 in the epithelial cell type. Primary nasal epithelial cells obtained from 2 healthy donors, and a human bronchial epithelial cell line 16HBE14o- expressed Spns2 localized to cytoplasm, nucleus, and cell membrane. In contrast to macrophage cell type, both primary nasal epithelial cells and the cell line 16HBE responded to cigarette smoke by a statistically significant decrease of Spns2 immunofluorescence (Fig 3).

### S1P export from cigarette smoke-exposed nasal epithelial cells

Relative levels of S1P inside and outside the cell were measured from THP-1 macrophages and primary cultures of nasal epithelial cells; a ratio between the extracellular and intracellular S1P would roughly reflect the rate of S1P export. While no significant changes of this ratio were detected in 3 experiments on THP-1 macrophages, for primary epithelial cells, 2 of 4 experiments showed statistically significant (p<0.05) decrease of the ratio in cigarette smoke extract-treated cells compared to untreated controls (S1 Fig).

### Spns2 in bronchial epithelium of cigarette smoke-exposed mice

**Fig 4:**
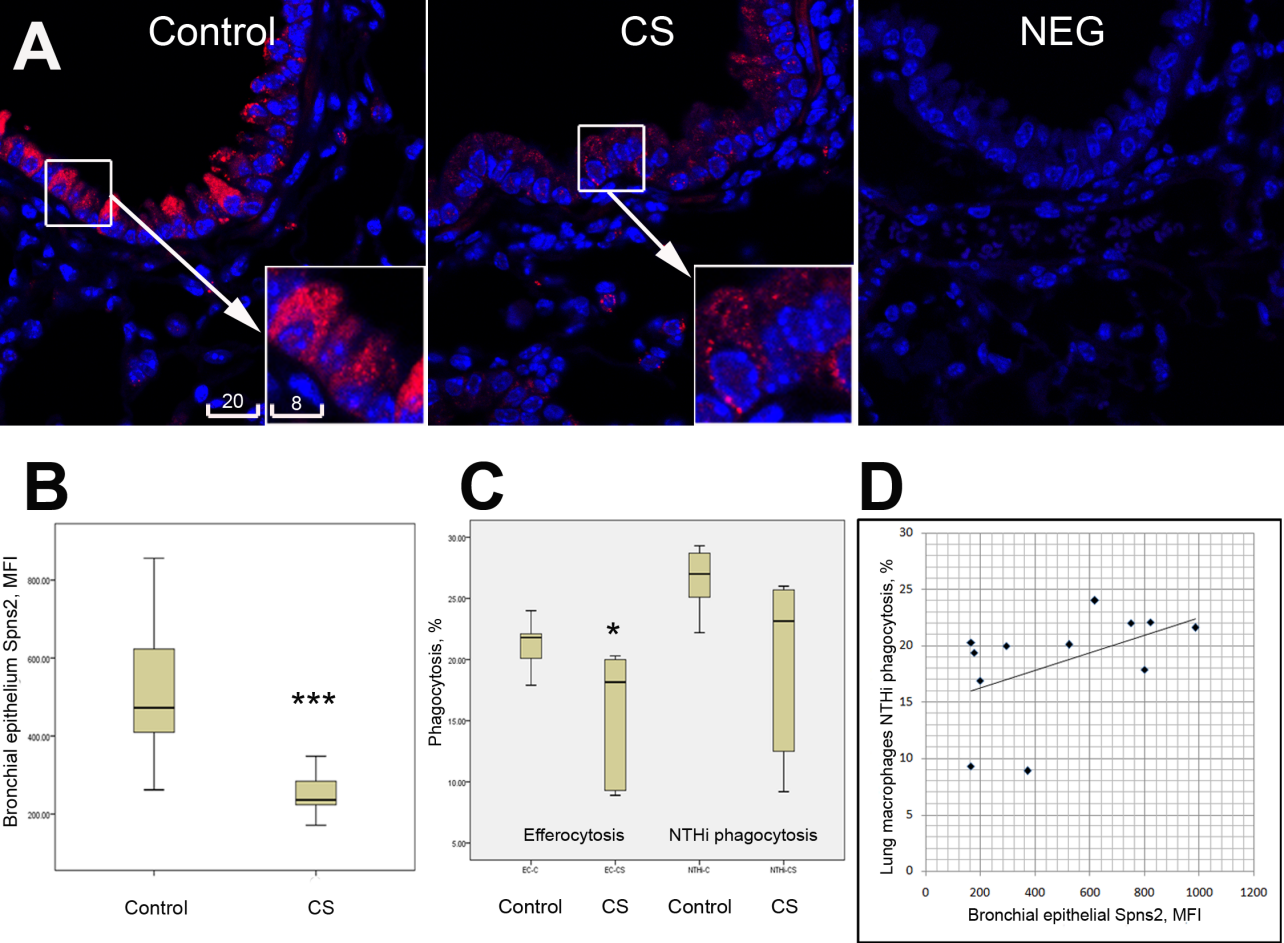
Defective phagocytosis in lung macrophages of cigarette smoked mice was associated with a down-regulated expression of Spns2 in bronchial epithelia. **A,** Representative confocal images of Spns2 in lung tissue of control and cigarette smoke (CS)-exposed mice, and a negative control staining (NEG). Spns2 was stained in red, nuclei in blue. Scale bars are in micrometers. **B,** Significant decrease of Spns2 (MFI, mean fluorescence intensity) in bronchial epithelia in CS-exposed vs. control mice (p<0.001, pooled data from 6 animals per group). **C,** Cigarette smoke exposure induced a significant decrease of efferocytosis (p<0.05, n = 6 per group) in lung macrophages of CS-exposed vs. control mice, and a nearly significant trend of reduced NTHi phagocytosis (p = 0.063). **D,** Correlation between NTHi phagocytic activity of pulmonary macrophages in individual mice and expression of Spns2 in their bronchial epithelia (pooled data from 12 mice, r = 0.685; p = 0.014 by Spearman’s rho test).

In a qualitative comparison, the brightest immunofluorescence of Spns2 in normal mouse lungs was localized to bronchial epithelia and type 2 alveolar cells, followed by alveolar macrophages which showed mostly nuclear staining, and lung vascular endothelia which showed dull fluorescence (S2 Fig). Whereas alveolar macrophages from cigarette smoke-exposed mice showed a non-significant trend for an increase of Spns2 expression (S2B Fig), a significant decrease of Spns2 immunofluorescence in bronchial epithelia was recorded (Fig 4A and 4B). Non-specific binding of secondary antibodies to mouse lung tissue sections was minimal (Fig 4A, negative control), and 2 primary antibodies to Spns2 raised in different species showed nearly identical patterns of staining (S2D Fig), which ruled out artefactual findings.

### Correlation between epithelial cell Spns2 expression and macrophage phagocytic capacity in cigarette smoke-exposed mice

In keeping with our previous studies in mouse [17, 18], chronic exposure to cigarette smoke induced a significant decrease in the capacity of pulmonary macrophages to phagocytose either apoptotic cells or NTHi (Fig 4C). A positive correlation between bronchial epithelial Spns2 immunofluorescence and NTHi phagocytosis was identified (Fig 4D).

## Discussion

Several lines of data have been presented in this study to support a novel hypothesis, that the inhibitory effects of cigarette smoke on macrophage phagocytic function in the airway may be associated with disruption of epithelial-macrophage crosstalk via intercellular S1P signaling involving Spns2.

Effective macrophage phagocytic capacity is key to the success of the bronchial epithelial repair process by clearing excess inflammatory cells and accumulated apoptotic epithelial cells (efferocytosis). In COPD we have reported an aberrant accumulation of apoptotic bronchial epithelial cells and a specific dysfunction in the ability of alveolar macrophages to efferocytose the apoptotic cells. The impaired efferocytosis was observed in lung and alveolar macrophages from both current and ex-smoker subjects with COPD and smokers. We showed that the uncleared material may undergo secondary necrosis with pro-inflammatory effects [6]. These defects may have important effects in the lung, including perpetuation of inflammation, infection, and tissue damage, and explain why, once COPD is established, smoking cessation alone does not entirely reverse the abnormal chronic airways inflammation. We also found a defect in the capacity of alveolar macrophages to phagocytose bacteria in COPD [7]. Increased bacterial colonization is common in COPD and is thought to contribute to chronic airway inflammation, exacerbations and to the progression of COPD [22]. Thus improving clearance of both apoptotic cells and bacteria in the airway may reduce the risk of secondary necrosis and consequent inflammation as well as contributing to a reduction in the rate of exacerbations in COPD. In this regard our subsequent research provided substantial evidence that the macrophage dysfunction is a novel target for therapeutic manipulation with clinical significance. We showed that therapies, including Azithromycin, improved efferocytosis and reduced airway epithelial cell apoptosis, oxidative stress, and inflammation in smoke-exposed mice and COPD subjects *in vivo* [8, 10]. Despite extensive research however, the exact causes of the phagocytic defect in COPD remain unknown and the mechanisms and regulation of efferocytosis have emerged as important areas of research in the pathogenesis of pulmonary disease.

S1P signaling is emerging as both a key mechanism and a potentially important therapeutic target in lung diseases [23]. As a pleiotropic molecule the effects of S1P are complex, involving both pro- and anti-inflammatory mechanisms. We have previously reported that exogenous S1P improved macrophage phagocytic function [14], and others have shown that extrusive removal of apoptotic bronchial epithelial cells was dependent on S1P export [24]. Exogenous S1P upregulated the pro-inflammatory COX2 pathway in airway smooth muscle cells [25; 26]; however, it was shown to downregulate COX2, MMPs and NFκB p65 in models of LPS-stimulated mouse monocyte-derived macrophages [27] or IL-1β-stimulated human chondrocytes [28]. The reasons for such opposing effects are unclear. S1P acts on both intracellular targets and membrane-bound S1P receptors. In the latter case, the autocrine/paracrine effects of S1P are regulated in a complex manner by its intracellular synthesis, transport across cell membrane, as well as cell type-specific expression of G-coupled S1P receptors (of 5 isoforms, ligating of which may result in opposite effects), and enzymes catalysts for S1P breakdown. Each of the listed components could therefore be a potential target for modulation of S1P signaling.

Spns2 is a membrane transport protein first described in 2009 [15]. The only known function of Spns2 is in transport of S1P [15], dihydro-S1P [29], and the phosphorylated form of the S1P analogue FTY720 [30], all of which are immune-regulators. Spns2 has been implicated in a variety of physiologic and pathologic processes, including hyperoxia-induced lung damage [31], airway inflammation and hypersensitivity [32] and non-small cell lung cancer [33]. Human lung was shown to express the highest level of Spns2 transcripts compared to other investigated organs [30]. Of various cell types, expression and functions of Spns2 in endothelial cells have been described [31, 34]; however, to our knowledge there have been no previous reports of Spns2 expression in macrophages and epithelial cells, or its links to pulmonary macrophage phagocytic function. We firstly showed an abundance of SPHK1/2 and Spns2 protein expression in primary human bronchial epithelial cells with the latter localized to the apical cilia; immunofluorescence of normal mouse lung for Spns2 indicated the brightest level in bronchial epithelium compared to alveolar macrophages and vascular endothelium. Furthermore, in both models of cigarette smoke exposure *in vitro* or *in vivo*, we showed a significant a decrease of Spns2 expression in epithelial cells, consistent with a decrease in the ratio between extracellular and intracellular levels of S1P. Importantly, the decreased expression of Spns2 in bronchial epithelium of smoke-exposed mice significantly correlated with the reduced macrophage phagocytic activity, supporting a potential role for disrupted epithelial/macrophage cross-talk and S1P transport via Spns2 in the defective macrophage function in COPD. In contrast to the epithelial cell type, primary alveolar macrophages and THP-1 macrophages were found to respond to cigarette smoke by an increase of Spns2 expression, which was consistent with the finding of Spns2 upregulation in alveolar macrophages from smokers/COPD patients. The biologic function of this response is unclear from this study, though it could be hypothesized as a feed-back response of macrophages to compensate for the shift of the sphingolipid rheostat toward favoring apoptosis and impairment of phagocytic function by an increase of ceramides in COPD/cigarette smoke exposure [35].

Taken together with our previous report of a cigarette smoke-induced inhibition of SPHK1 activity and an exogenous S1P-facilitated improvement in phagocytic function in THP-1 macrophages, our data suggest that the down-regulation of Spns2 and consequent decrease of S1P export from the bronchial epithelium in COPD and in response to cigarette smoke may affect alveolar macrophage phagocytic activity via insufficient S1P receptor signaling. Further studies including primary cell co-culture systems are now designed to explore the direct biological effects of differential expression of Spns2 by alveolar macrophages and bronchial epithelial cells and how cigarette smoke- or COPD disease-induced changes of other molecules or factors might affect Spns2 expression, S1P release and related macrophage phagocytic dysfunction.

A potential limitation of our study is the relatively small numbers in some groups; however, despite this we were able to support our hypothesis by showing a significant increase in Spns2 immunofluorescence in COPD alveolar macrophages compared to those from non-COPD patients and healthy controls, and a significant correlation between epithelial expression of Spns2 and macrophage phagocytic capacity in mouse. Further studies are warranted to examine cytoplasmic and nuclear localization of Spns2 in macrophages to better understand the intracellular functions of this protein. Other possible consequences of cigarette smoke-induced down-regulation of Spns2 and reduced S1P export in the airway epithelium also remain to be determined.

In conclusion, we described a differential expression of Spns2, its subcellular distribution and response to cigarette smoke exposure, between airway epithelial cells and alveolar macrophages. Our data suggest that the epithelium may be the major source for extracellular S1P in the airway and that there is a possible disruption of epithelial/macrophage cross talk via S1P signaling in COPD and in response to cigarette smoke exposure. These findings identify a potential target for therapeutic advances in COPD.

## Supporting information

S1 Table Donors’ demographic data. (DOCX)

S1 Text Predicted bi-partile nuclear localization sequence in human Spns2. (DOC)

S1 Fig Cigarette smoke extract-induced decreased extracellular/intracellular ratio of S1P levels in human primary nasal epithelial cells. (TIF)

S2 Fig Immunofluorescence of Spns2 in mouse lung tissue. (TIF)
