# Supplementary material for: Disrupted epithelial/macrophage crosstalk via Spinster homologue 2-mediated S1P signaling may drive defective macrophage phagocytic function in COPD
Source: PLoS One. 2017 Nov 7;12(11):e0179577. doi: 10.1371/journal.pone.0179577 (PMC5675303; doi:10.1371/journal.pone.0179577)
Supplement: S1 Table — (DOCX) [file pone.0179577.s001.docx]

S1 Table. Donors demographic data

| *Patients ID* | *Gender* | *Age* | *Conditions* | *Spns2 MFI* |
| --- | --- | --- | --- | --- |
| 1A | M | 63 | Control | 20.2 |
| 1B | F | 19 | Control | 39.1 |
| 1C | M | 34 | Control | 29.1 |
| 1D | M | 19 | Control | 34.2 |
| 1E | M | 19 | Control | 30.0 |
| 1F | M | 24 | Control | 28.0 |
| 1G | M | 66 | Control | 60.1 |
| 1H | F | 49 | Control | 40.8 |
| 2A | M | 62 | Smoker nonCOPD | 76.2 |
| 2B | M | 58 | Smoker COPD | 57.1 |
| 2C | M | 83 | Smoker COPD | 89.6 |
| 2D | M | 55 | Smoker COPD | 68.4 |
| 2E | M | 76 | Smoker COPD | 73.6 |
| 3A | M | 51 | Transplant | 22.8 |
| 3B | M | 67 | Transplant | 39.9 |
| 3C | M | 57 | Transplant | 45.2 |
| 3E | M | 67 | Transplant | 52.2 |
