# Supplementary material for: Disrupted epithelial/macrophage crosstalk via Spinster homologue 2-mediated S1P signaling may drive defective macrophage phagocytic function in COPD
Source: PLoS One. 2017 Nov 7;12(11):e0179577. doi: 10.1371/journal.pone.0179577 (PMC5675303; doi:10.1371/journal.pone.0179577)
Supplement: S1 Text — (DOC) [file pone.0179577.s002.doc]

| **Predicted bi-partile nuclear localization sequence in human Spns2, by the NLS Mapper software.**  [**http://nls-mapper.iab.keio.ac.jp/cgi-bin/NLS_Mapper_form.cgi**](http://nls-mapper.iab.keio.ac.jp/cgi-bin/NLS_Mapper_form.cgi) |
| --- |
| **MMCLECASAAAGGAEEEEADAERRRRRRGAQRGAGGSGCCGARGAGGAGV 50  SAAGDEVQTLSGSVRRAPTGPPGTPGTPGCAATAKGPGAQQPKPASLGRG 100  RGAAAAILSLGNVLNYLDRYTVAGVLLDIQQHFGVKDRGAGLLQSVFICS 150  FMVAAPIFGYLGDRFNRKVILSCGIFFWSAVTFSSSFIPQQYFWLLVLSR 200  GLVGIGEASYSTIAPTIIGDLFTKNTRTLMLSVFYFAIPLGSGLGYITGS 250  SVKQAAGDWHWALRVSPVLGMITGTLILILVPATKRGHADQLGDQLKART 300  SWLRDMKALIRNRSYVFSSLATSAVSFATGALGMWIPLYLHRAQVVQKTA 350  ETCNSPPCGAKDSLIFGAITCFTGFLGVVTGAGATRWCRLKTQRADPLVC 400  AVGMLGSAIFICLIFVAAKSSIVGAYICIFVGETLLFSNWAITADILMYV 450  VIPTRRATAVALQSFTSHLLGDAGSPYLIGFISDLIRQSTKDSPLWEFLS 500  LGYALMLCPFVVVLGGMFFLATALFFVSDRARAEQQVNQLAMPPASVKV 549** |
