# Supplementary figures and images for: Disrupted epithelial/macrophage crosstalk via Spinster homologue 2-mediated S1P signaling may drive defective macrophage phagocytic function in COPD
Source: PLoS One. 2017 Nov 7;12(11):e0179577. doi: 10.1371/journal.pone.0179577 (PMC5675303; doi:10.1371/journal.pone.0179577)

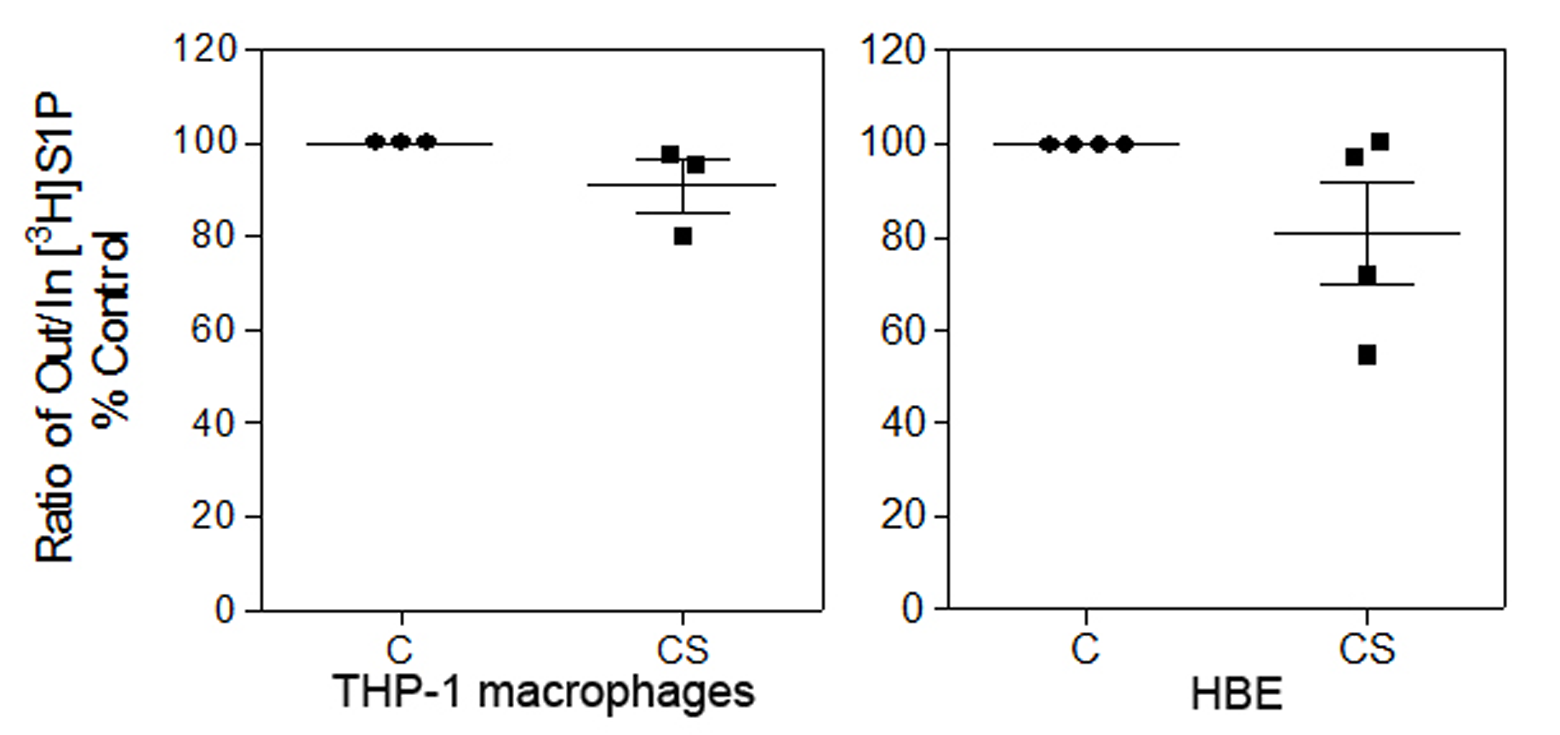

Supplement: S1 Fig — (TIF) [file pone.0179577.s003.tif]

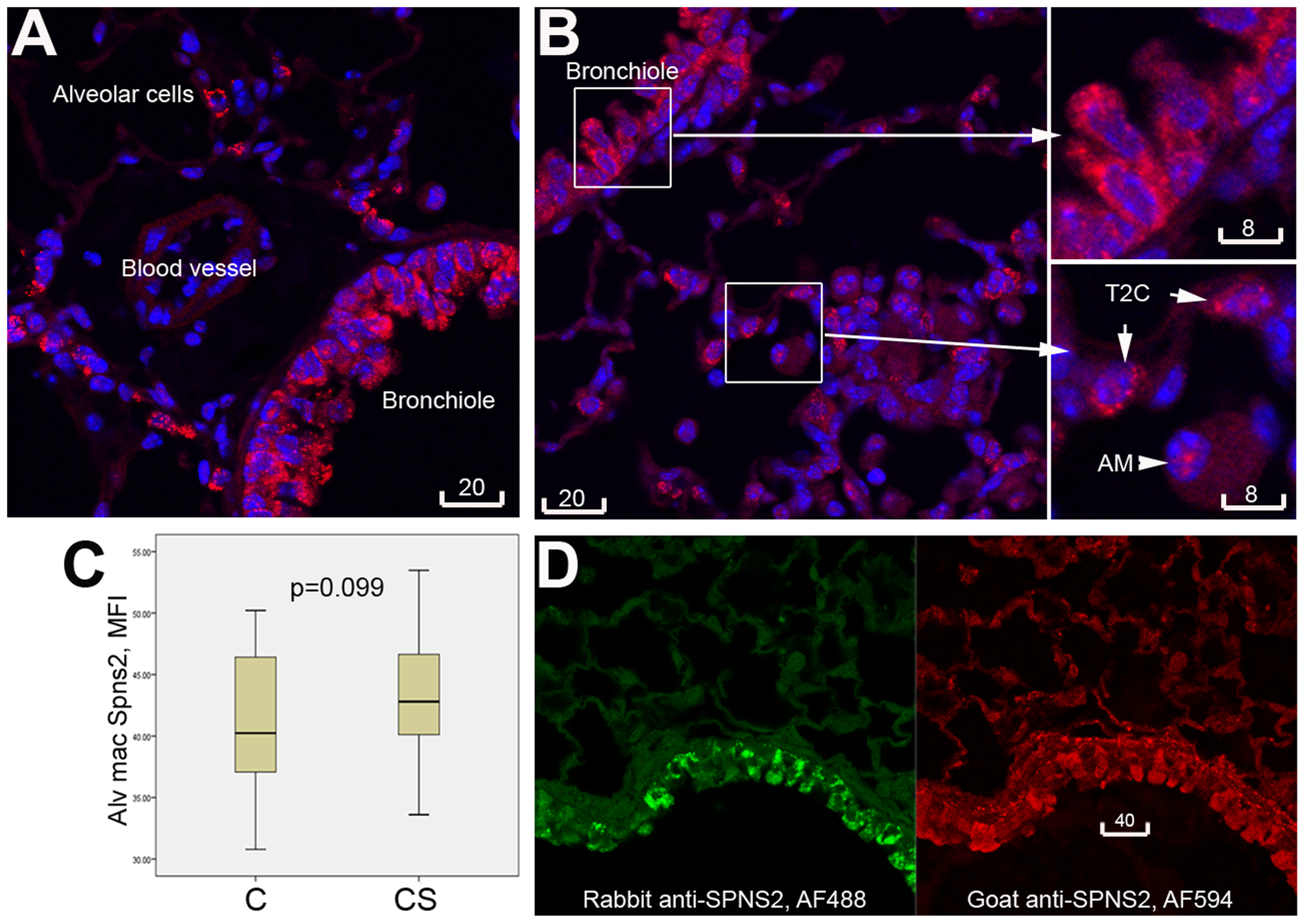

Supplement: S2 Fig — (TIF) [file pone.0179577.s004.tif]
